# A comparison of two cleaning methods applied in a small animal hospital

**DOI:** 10.1186/s12917-025-04631-0

**Published:** 2025-03-15

**Authors:** Todd Alsing-Johansson, Elin Torstensson, Karin Bergström, Susanna Sternberg-Lewerin, Anna Bergh, Johanna Penell

**Affiliations:** 1 Department of Clinical Sciences, Faculty of Veterinary Medicine and Animal Science, Swedish University of Agricultural Sciences, Uppsala, 750 07 Sweden; 2 Uppsala Veterinärklinik Evidensia, Evidensia Djursjukvård, Danmarksgatan 26, Uppsala, 753 23 Sweden; 3 Department of Animal Health and Antimicrobial Strategies, Swedish Veterinary Agency, Uppsala, 751 89 Sweden; 4 Department of Animal Biosciences, Faculty of Veterinary Medicine and Animal Science, Swedish University of Agricultural Sciences, Uppsala, 750 07 Sweden

**Keywords:** Bacterial reduction, Biosecurity, Contamination, Disinfection, Healthcare-associated infection, Hygiene, Infection prevention and control, Veterinary clinic

## Abstract

**Background:**

Environmental cleaning of near-patient surfaces in animal healthcare is an important infection prevention and control measure to lower the risk of spread of healthcare-associated infections (HAIs). There is a lack of reports on the effect of cleaning of near-patient surfaces in animal hospital wards. The aims of this study were to (1) determine bacterial load before cleaning, on near-patient surfaces in dog cages in a mixed medical and surgical ward and investigate factors associated with this bacterial load (2) compare the bacterial reduction on these surfaces after cleaning with (a) a scrubbing brush with detergent and rinsing before and after cleaning, and (b) a microfibre mop moistened with water, and after disinfection carried out after each cleaning method. In each cage the floor and the wall were sampled before cleaning, after cleaning, and after disinfection. Bacterial load and reduction were log_10_-transformed and for comparisons t-test, one-way Anova and Wilcoxon rank sum test were used. A generalized additive model was performed for analysis of the association between factors and bacterial load.

**Results:**

The bacterial load in dog cages before cleaning varied, higher loads were noted after longer stay in the cage. The bacterial reduction was in most cases more effective after cleaning with scrubbing brushes with detergent compared to cleaning with damp microfibre mops. After cleaning, a majority of the samples were below the suggested threshold value 2.5 CFU/cm^2^, except for floor samples after microfibre cleaning. No significant difference in bacterial load, between cleaning methods was noted after disinfection. Overall, the bacterial load was significantly lower on walls than on floors.

**Conclusions:**

Overall, the bacterial load was below the suggested threshold value after decontamination, except after microfibre cleaning of the floor. Scrub cleaning with a detergent should be considered for cleaning of anti-slip surfaces like the cage floor. The study shows a need for evidence-based cleaning and disinfection routines for near-patient surfaces and evidence-based threshold values for bacterial load, to reduce the risk of HAIs.

**Supplementary Information:**

The online version contains supplementary material available at 10.1186/s12917-025-04631-0.

## Background

In human healthcare, environmental microorganisms on near-patient surfaces pose a risk of healthcare-associated infections (HAIs) [1–3] and presumably this also applies to animal healthcare [4, 5]. In animal healthcare HAIs have been reported to lead to longer hospital stays, increased healthcare costs, morbidity and mortality [6–8]. Environmental cleaning and disinfection of near-patient surfaces is an important infection prevention and control (IPC) measure to limit the amount of pathogenic microorganisms in animal and human healthcare facilities, and to reduce the risk of HAIs [3, 9].

Studies of bacterial load on near-patient surfaces in dog cages in animal healthcare facilities are lacking. Furthermore, for animal healthcare, there are no suggested threshold values for bacterial load on near-patient surfaces. The suggested threshold values for total bacterial load for near-patient surfaces in human healthcare are often below 2.5 and sometimes below 5 colony forming units (CFU)/cm^2^ [10–13]. Still, there are no studies in animal or human healthcare showing evidence of a reduced risk of HAIs if the suggested threshold values are reached after cleaning and/or disinfection.

In Sweden, cleaning practices of patient cages in small animal healthcare, commonly involve use of a microfibre mop moistened with water, but without detergent. This has replaced the earlier common cleaning method which included rinsing, application of a detergent, followed by scrubbing with a scrubbing brush or mop, rinsing again and finally removing water with a squeegee. The use of microfibre cloths and damp mops for cleaning is likely due to a desire to lower the environmental impact from detergents, to reduce the risk of aerosol spread of microorganisms, and to save time [14].

Beside the use of damp microfibre mops, damp microfibre cloths are also commonly used in Sweden, e.g. for surface cleaning of e.g. examination tables in examination rooms between patients instead of previous practice, i.e. disinfection with a wiping paper moistened with an alcohol and surfactant. There are several methods for cleaning environmental surfaces that have been studied in laboratory studies and in human healthcare. Moore and Griffith [15] showed in a laboratory study that microfibre cloths of different brands moistened with water, differed in effect when used for damp cleaning on surfaces, from a small increase to a log_10_ 2 reduction and between a log_10_ 0.6 and log_10_ 1.6 reduction of soil. Only one of the six tested microfibre cloth brands removed soil significantly better than a damp paper towel [15]. Damp ultramicrofibre cloths, a thinner fibre than the standard microfibre, successfully reduced bacterial load on laminate, steel, smooth (vinyl) and rough (linoleum) tiles in a laboratory study [16]. Another laboratory study showed that cleaning with microfibre cloths with detergent/disinfectant reduced the bacterial load significantly more than cleaning with microfibre cloths and water [17]. Using a microfibre cloth for cleaning of more than one surface, in laboratory studies, resulted in cross-contamination from one surface to the next [15, 18]. In a human healthcare study, cleaning with microfibre mops with a detergent reduced the total bacterial load more effectively on the floor compared to cleaning with cotton string mops with a detergent [19]. Cleaning of floors with a scrubbing machine, followed by rinsing and drying, was more effective in human healthcare in reducing coagulase-positive staphylococci compared to vacuuming followed by damp mopping with a cotton or mixed fibre mop with detergent [20]. However, there was no significant difference between the cleaning methods in reduction of total bacterial load [20]. Lack of similar studies in animal healthcare facilities and the fact that current human healthcare studies differ in result limits the ability for evidence-based decisions on what cleaning methods and products to use in animal healthcare.

The aims of this study were to.

1. determine bacterial load before cleaning on near-patient surfaces in dog cages in a small animal hospital ward and investigate factors associated with this bacterial load, and
2. compare the bacterial reduction on these surfaces after cleaning with (a) a scrubbing brush with detergent and rinsing before and after cleaning, and (b) a microfibre mop moistened with water, and after disinfection carried out after each cleaning method.

## Methods

### Study design

The study was an experimental, randomized study with a parallel group design. It was carried out in the ward of a university small animal hospital in Sweden during spring 2022. The total patient load consists of approximately 23 000 patients per year, of which 1900 patients per year were admitted to the mixed medical and surgical ward. There has been occasional isolation of bacteria associated with HAI, such as MRSA and MRSP but this is not common and there are routines in place for prevention and control. Prior to the study a pilot study was carried out to test the cleaning, disinfection, and sampling protocols. Sample size was calculated to estimate the number of cages (23 in each group) needed to be able to find a clinical relevant difference in bacterial reduction between cleaning methods.

### Cages

**Fig. 1:**
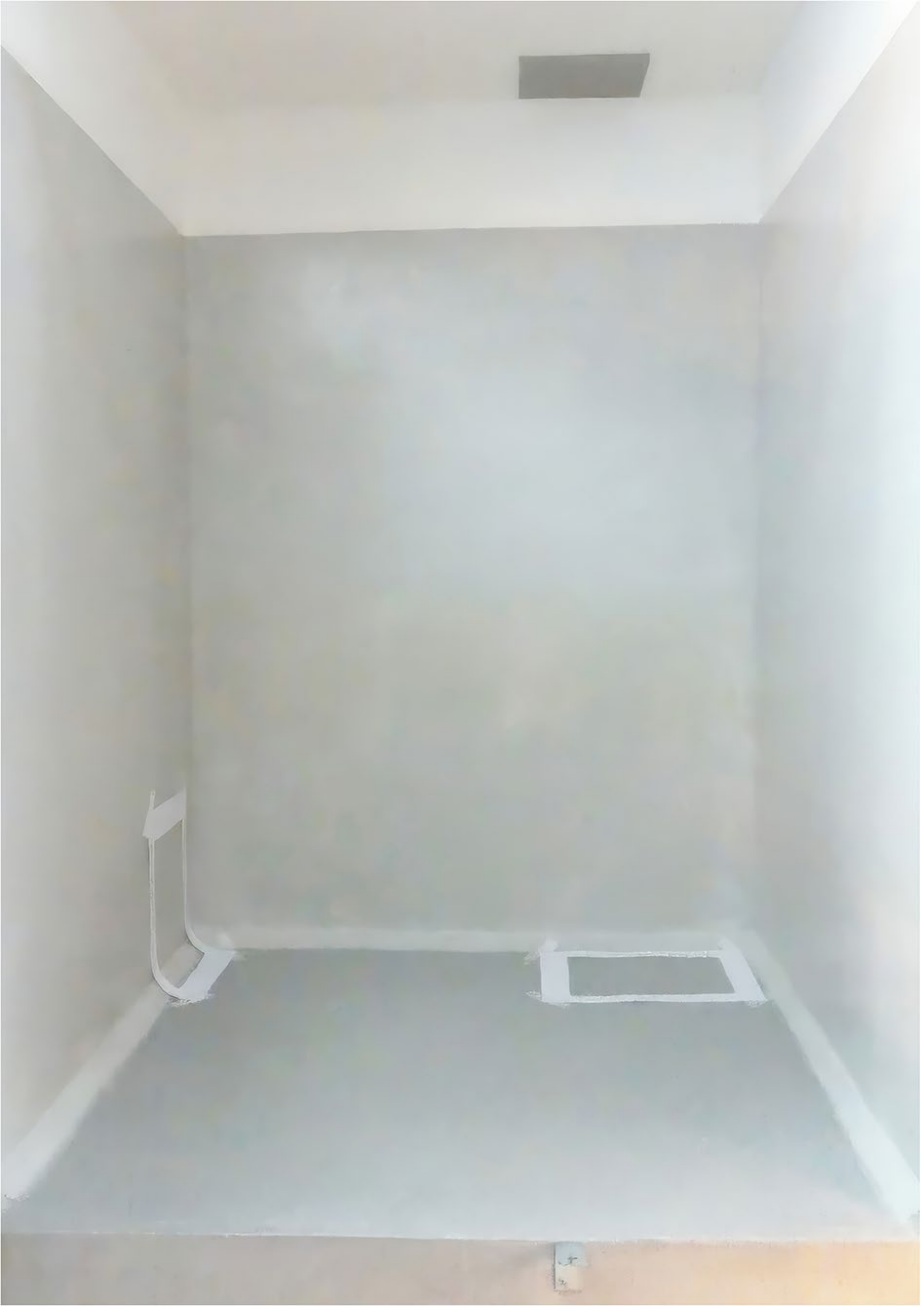
Example of one of the dog cages cleaned and disinfected in the study

Four ward rooms with six cages in each room were included in the study. The cages are used for small and middle-sized dogs, and they measure 115 cm (depth) * 120 cm (width) * 180 cm (height), see Fig. 1. The surface material on the floor is epoxy with an anti-slip additive (quarts 0.7–1.2 mm) (Peran STB Struktur, Flowcrete, Tremco CPG Sweden AB, Gothenburg, Sweden). The floor has a low slip potential, the slip resistance on both dry and wet floor is > 40 (BS 7976-2). The surface material on the wall is epoxy. Patients at the small animal hospital ward are often hospitalized for a shorter period, usually they only stay for one or two days.

### Inclusion and exclusion criteria

Cages in need of cleaning were inspected by the first and second author to identify cages meeting the inclusion criteria. Cages visibly clean or with some minor stains were included in the study. Heavily soiled cages with e.g. faeces, urine, blood or feed residues were excluded, since heavily soiled surfaces require a more thorough cleaning than the standardised cleaning protocol used in this study. Cages used for less than 12 h were excluded from the study, to assure the dog had stayed in the cage for some time even if the visit included various examinations and/or surgery. Cages that had been empty for up to four days before cleaning were included in the study since cages were generally cleaned within four days after usage in the animal hospital. Totally 46 dog cages were included in the study, 23 with each cleaning method.

### Randomization

Each sampling day the cages were randomized, using a lottery which cleaning method to use, half of the cages to each method.

### Cleaning

**Fig. 2:**
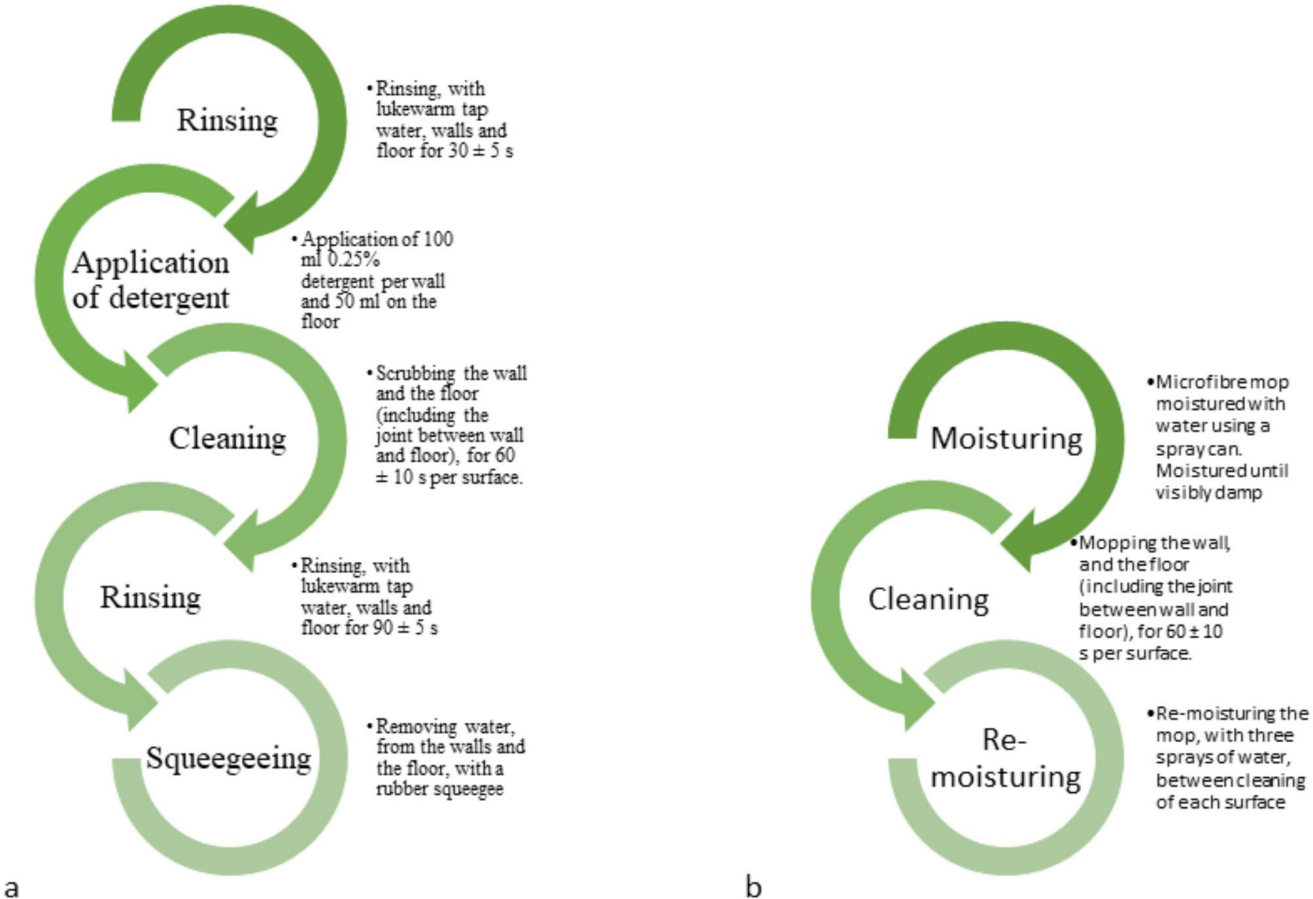
Cleaning protocol for the two cleaning methods used in the study, (**a**) Scrub cleaning and (**b**) microfibre cleaning

All cleaning and disinfection were carried out by the second author. The procedure began with the three walls starting at the highest point, cleaning downwards to the floor, continued with the joint between wall and floor and finally the floor was cleaned. Cleaning was carried out for a specific time (Fig. 2) per wall and floor. After the procedure was finished it was inspected that all surfaces were visibly clean. Detailed cleaning protocols can be found in Fig. 2. After cleaning the surfaces were left to dry before the disinfection.

Two cleaning methods were compared in the study:

- A standardized version of the studied animal hospital’s cleaning protocol: rinsing with lukewarm tap water, cleaning with a scrubbing brush (Deck Scrub, waterfed, 270 mm, Very hard, White, Vikan A/S, Skive, Denmark) and a 0.25% detergent (Allotol Natur, Nordexia AB, Bromma, Sweden), rinsing with lukewarm tap water, and finally removing the water with a rubber squeegee (MultiSqueegee 35 mm, Vileda Professional, Freudenberg Home and Cleaning Solutions AB, Norrköping, Sweden) (further called “scrub cleaning”) and
- Cleaning with a microfibre mop (Duotex^®^ Shine Plus Mop 30 cm, Micro System Duotex AB, Solna, Sweden) moistened with tap water (further called “microfibre cleaning”).

The scrubbing brushes were new and previously unused. Before the first use, the scrubbing brushes were rinsed, disinfected at 90 °C, and dried in a washer-disinfector (WD14 Tablo, Getinge, Gothenburg, Sweden), and finally sterilized at 121 °C in an autoclave (HS-22 K7+, Getinge, Gothenburg, Sweden). One scrubbing brush was used for cleaning one cage. After each use, the scrubbing brush was first rinsed with tap water just outside the cage, and then rinsed, disinfected at 90 °C and dried in a washer-disinfector. The scrubbing brushes were then air-dried on disposable sheets (Abri-Soft Disposable Sheets, Abena A/S, Aabenraa, Denmark) on a bench in a closed room where instruments and equipment were cleaned, disinfected and sterilized. When they were dry, they were stored in a closed plastic box for up to seven days. The plastic box was disinfected with isopropanol added with surfactant (DES + 45, Liv By Clemondo, Helsingborg, Sweden) before it was used for storage of scrubbing brushes. If left for more than seven days, the scrubbing brushes were re-processed in the washer-disinfector before use. The scrubbing brush handle and the rubber squeegee were disinfected using wiping paper moistened with isopropanol added with surfactant between each cage.

The microfibre mops were new and never used before the start of the project. One microfibre mop was used for cleaning one cage. Between each use the microfibre mops were cleaned separately at 95 °C in a washing machine (Electrolux W4180H, Electrolux Professional, Stockholm, Sweden) without fabric softener and tumble dried (Electrolux T5350, Electrolux Professional, Stockholm, Sweden) at low heat, until dry and left in a closed plastic box. The plastic box was disinfected with isopropanol added with surfactant. If left for more than seven days, the microfibre mops were re-cleaned and dried before use. Mop handles were disinfected using wiping paper moistened with isopropanol added with surfactant between each cage.

Additional variables related to sampling and cleaning methods were collected as follows; time patient spent in the cage and time the cage was empty before cleaning. The variables effect on the bacterial load before cleaning was analysed, see section data analysis.

### Disinfection

After both cleaning methods, the dry walls and floor were disinfected with a potassium monosulphate (DesiDos™, SeptiChem ApS, Holte, Denmark) disinfectant, diluted according to the producer’s instruction. A volume of 3 dl disinfectant was applied first on the walls and then on the floor and 0.5 dl on a mop (Vileda UltraMax Refill, Vileda, Freudenberg Home and Cleaning Solutions GmbH, Weinheim, Germany) of blended material. In total 3.5 dl ± 0.5 dl disinfectant was used per cage. One mop was used for application of the disinfectant on the walls and the floor in one cage. The mops were washed according to the studied animal hospital’s routines, together with other laundry from the medical and surgical ward, at 60–95 °C. The mops were hang-dried in a corridor outside the washing room. When dry they were kept in a drawer with other types of mops.

### Sampling

**Fig. 3:**
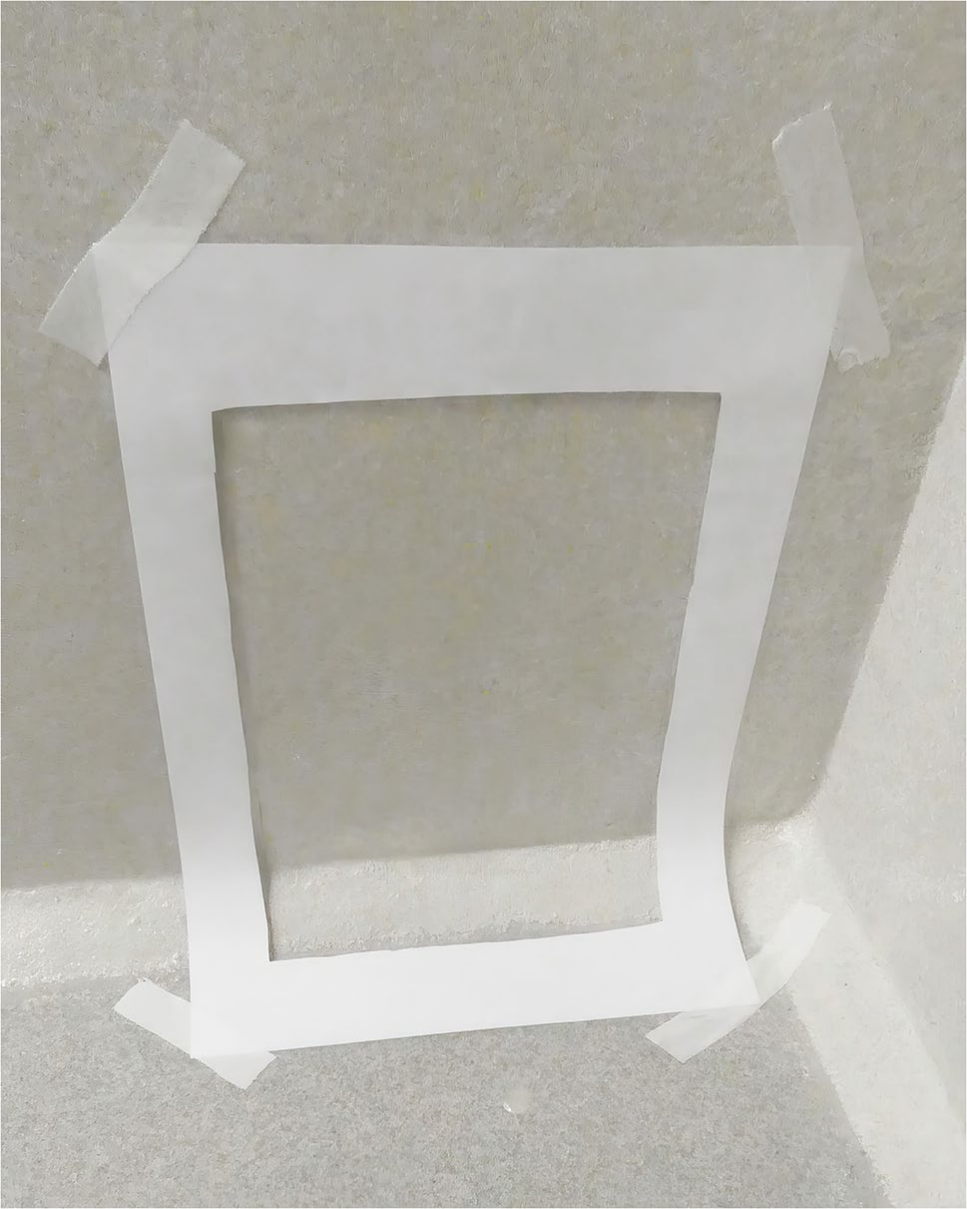
Sampling frame on the wall

**Fig. 4:**
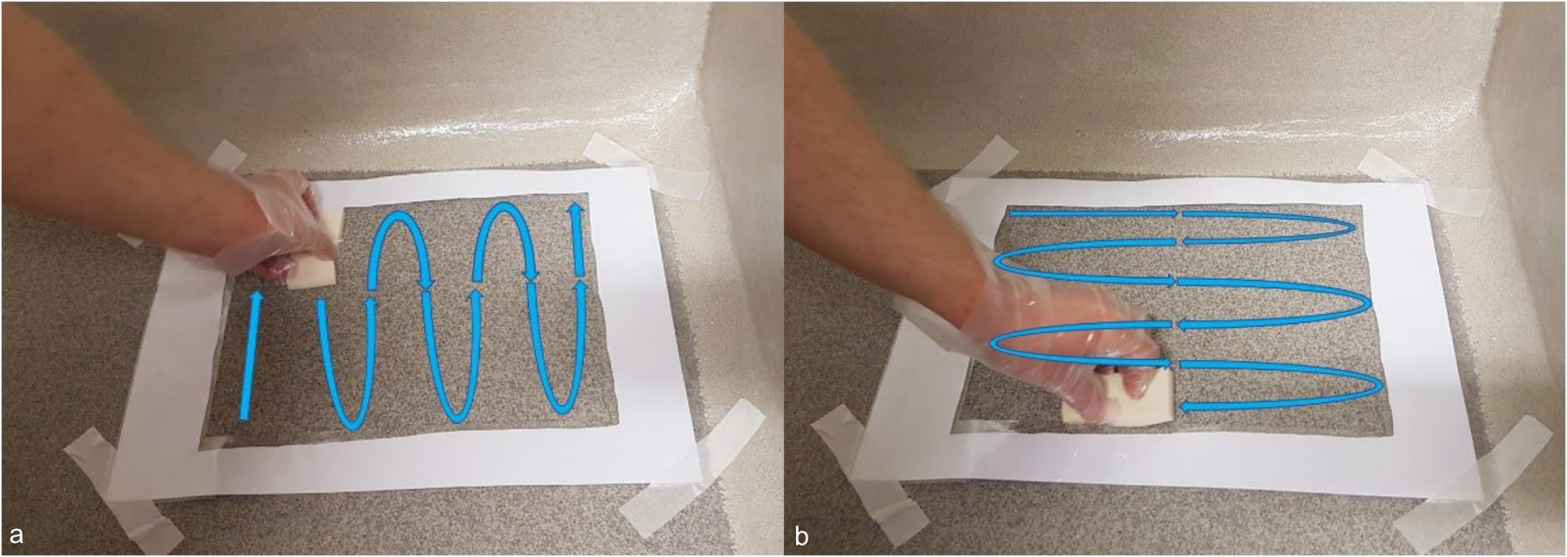
Sampling of the floor with the sampling sponge

A total number of 276 bacteriological samples were taken in the study. All sampling was carried out by the first author. A sampling sponge (SampleRight™ sponge sampler, World Bioproducts, Libertyville, Illinois, USA) in sterile single sample bag with 10 ml neutralizing broth (HiCap neutralizing broth, World Bioproducts, Libertyville, Illinois, USA) was used for sampling. The sampling sponge was handled with sterile gloves (Examination gloves, sterile, pair-packed, Mediplast, Malmö, Sweden) which were changed between each sampling, using an aseptic technique. When needed hands were cleaned and/or disinfected, according to hand hygiene routines. Sampling was performed before cleaning, after cleaning (in median ~ 3 h after cleaning), and after disinfection, all on dry surfaces. Sampling was carried out in the same place and in the same way in all cages using a frame of laminated paper cut out of an A3 paper, with an inner and outer board of laminated plastic around the paper frame, and fixated with textile tape (Durapore, 2.5 cm, 3 M, Minneapolis, Minnesota, USA) (Fig. 3). The size of the sampled area was 21.0*29.7 = 623.7 cm^2^ (an A4 paper). The surface sampled was first swabbed vertically and then horizontally (Fig. 4). The wall sample included the joint between wall and floor since that was suspected to be the most difficult part of the wall to clean (Fig. 3). The frame was disinfected using a wiping paper moistened with isopropanol added with surfactant before each sampling. Samples was kept in a cooling bag, before taken to the laboratory where they were kept in a refrigerator (4–8 °C) until analysed. Samples were taken to the laboratory continuously during a sampling day. For every sampling day a negative control, a sealed sampling sponge, was kept on top of the cooling bag until the last sample was put in the bag. On the first sampling day, including 36 samples, the laboratory analyses started the same day as the sampling. During the rest of the sampling period analyses started the day after sampling. According to the manufacturer, the sampling sponge can be kept refrigerated for up to 72 h before start of analyses [21].

### Bacteriological analyses

Bacteriological analyses were carried out by the first author at the bacteriological laboratory at The Swedish University of Agricultural Sciences. All samples were homogenized in a stomacher (Easy MIX Lab Blender, AES-Chemunex, Weber Scientific, Hamilton, New Yersey, USA) for 120 s at 240 rpm. Subsequently, the samples were 10-fold serial diluted in Maximum Recovery Dilutent (MRD) (Dilucup^®^ Elegance MRD, LabRobot Products AB, Stenungsund, Sweden). Samples taken before cleaning were diluted: 1:10, 1:100 and 1:1000, samples taken after cleaning were diluted 1:1, 1:10 and 1:100, and samples taken after disinfection were diluted 1:1. One ml of each dilution was incubated on Petrifilm™ AC plate (3 M™ Petrifilm™ Aerobic Count Plate, Saint Paul, Minnesota, USA). The petrifilms were incubated in 37 °C ± 1 °C for 48 h ± 2 h. The number of colony-forming units (CFU) was counted manually, preferably on Petrifilms™ with 20–250 CFU. For each sample, CFU/cm^2^ was calculated using a standard formula (ISO 7218:2007). Bacterial load was quantified as < 2.5 CFU/cm² = very scanty growth, 2.5–4.9 CFU/cm² = scanty growth, 5.0–12.0 CFU/cm² = light growth, 12.1–40.0 CFU/cm² = moderate growth, 40.1–100.0 CFU/cm² = heavy growth, and > 100.0 CFU/cm² = very heavy growth modified from Dancer [13], Griffith et al. [12], Lewis et al. [10], Mulvey et al. [11], and White et al. [20].

### Data analysis

Microsoft^®^ Excel 2016 (16.0.5134.1000) (Microsoft Corporation, Redmond, Washington, USA) was used for data management and descriptive statistics. Bacterial loads and reductions were log_10_-transformed. A few samples showed 0 CFU after disinfection of the wall, both cleaning methods. To enable log_10_-transformation this was handled by adding a small offset (c), equal to half the smallest positive value (c = 0.5) [22]. Bacterial load and effect of cleaning and disinfection between surfaces and methods were compared using Welch two sample t-test, one-way Anova or Wilcoxon rank sum test.

Spearman’s rank correlation was performed for analysis of correlation between bacterial load on the floor and the wall before cleaning. A generalized additive model (GAM) was performed for analysis of the relationship between thebacterial load on the floor (log10(load_floor)) before cleaning and the potential explanatory factors time patient spent in the cage (days_used), time the cage was empty before cleaning (days_empty) and the bacterial load on the wall (log10(load_wall)). The model was adjusted with spline effect (s) and interaction (by) so that the highest deviance possible was explained without inflated standard errors. The script in the final GAM model was: log10(load_floor) ~ s(log10(load_wall)) + s(log10(load_wall), by = days_used) + s(log10(load_wall), by = days_empty).

Residual plots and Shapiro-Wilk normality test were used to evaluate normality. As significance level 0.05 was used. Statistical analyses were performed in RStudio version 2021.9.0.351 (RStudio Team (2021). RStudio: Integrated Development Environment for R. RStudio, PBC, Boston, MA), packages ggplot2 (v3.4.3; [23]), mgcv (v1.8-36; [24]) emmeans (v. 1.8.9 [25]) and predictmeans (v. 1.0.9 [26]).

## Results

### Bacterial loads on floors and walls

The median bacterial load before cleaning was 9 CFU/cm^2^ on the floors and 2.6 CFU/cm^2^ on the walls, 15% of all floor samples were below 2.5 CFU/cm^2^ and 48% of all wall samples were below 2.5 CFU/cm^2^ (see Tables 1 and 2). There was no significant difference in bacterial load before cleaning between cages cleaned with the different protocols. The bacterial loads were significantly lower on both floors and walls after scrub cleaning compared to microfibre cleaning. After scrub cleaning of the floors, 70% of the samples were below 2.5 CFU/cm^2^ compared to 35% of the samples after microfibre cleaning. After scrub cleaning of the walls, all samples were below 2.5 CFU/cm^2^ compared to 70% of the samples after microfibre cleaning. After disinfection, regardless of cleaning method, 78% of all floor samples were below 2.5 CFU/cm^2^ and 98% of all wall samples were below 2.5 CFU/cm^2^. The bacterial load on the walls was significantly lower than on the floors, before and after cleaning as well as after disinfection.

**Table 1:** Bacterial load reported as CFU/cm^2^ on the floors and walls before cleaning, after cleaning and after disinfection. Twenty-three cages were sampled per cleaning method

Surface	Sample	Median	Interquartile range	Range
Floor	Before scrub cleaning	6.8	3.3–25.3	1.1-320.7
Floor	Before microfibre cleaning	9.3	5.0–18.0	0.9-12826.7
Floor	After scrub cleaning	1.3	0.8–2.9	0.0-15.6
Floor	After microfibre cleaning	3.7	1.8–9.5	1.1-203.6
Floor	After scrub cleaning and disinfection	0.6	0.4–1.9	0.1-8.0
Floor	After microfibre cleaning and disinfection	0.8	0.4–1.7	0.2–16.2
Wall	Before scrub cleaning	2.8	1.7–6.8	0.7-133.1
Wall	Before microfibre cleaning	2.5	1.3–6.4	0.6–32.2
Wall	After scrub cleaning	0.2	0.1–0.6	0.0-1.5
Wall	After microfibre cleaning	0.7	0.4–2.5	0.3–17.3
Wall	After scrub cleaning and disinfection	0.4	0.0-0.6	0.0-1.8
Wall	After microfibre cleaning and disinfection	0.0	0.0-0.2	0.0-2.9

CFU = colony-forming units

**Table 2:** Distribution of CFU/cm² per sampling. Twenty-three cages were sampled per cleaning method

	< 2.5	2.5–4.9	5.0–12.0	12.1–40.0	40.1–100.0	> 100.0
	CFU/cm^2^	CFU/cm^2^	CFU/cm^2^	CFU/cm^2^	CFU/cm^2^	CFU/cm^2^
**Scrub cleaning floor**						
Before cleaning	3	7	2	7	3	1
After cleaning	16	3	3	1	0	0
After cleaning and disinfection	17	4	2	0	0	0
**Microfibre cleaning floor**						
Before cleaning	4	2	8	6	1	2
After cleaning	8	6	7	1	0	1
After cleaning and disinfection	19	2	1	1	0	0
**Scrub cleaning wall**						
Before cleaning	11	5	4	1	1	1
After cleaning	23	0	0	0	0	0
After cleaning and disinfection	23	0	0	0	0	0
**Microfibre cleaning wall**						
Before cleaning	11	4	5	3	0	0
After cleaning	16	4	2	1	0	0
After cleaning and disinfection	22	1	0	0	0	0

CFU = colony forming units

### Effect of cleaning, comparison between cleaning methods

When the bacterial load on the floors was between 5 and 40.0 CFU/cm^2^, scrub cleaning resulted in a significant larger reduction of the bacterial load compared to microfibre cleaning while there was a non-significant tendency towards larger reduction for scrub cleaning also for bacterial loads below 5 CFU/cm^2^, see Table 3. No difference in bacterial reduction on the floors was seen between the two cleaning methods when the bacterial load before cleaning was above 40.0 CFU/cm^2^. Regardless of the bacterial load on the walls before cleaning, scrub cleaning resulted in a significantly larger bacterial reduction compared to microfibre cleaning. The bacterial reduction, on both floors and walls, varied between − 0.3 and 3.4 log_10_ CFU/cm^2^ for scrub cleaning and − 1.1 and 3.0 log_10_ CFU/cm^2^ for microfibre cleaning. In one case there was a 1.1 log_10_ increase, from 15 CFU/cm^2^ to 204 CFU/cm^2^, in bacterial load after microfibre cleaning of the floor.

**Table 3:** Stratified comparison of bacterial reduction between two cleaning methods and cleaning effect within method and surface

Surface	Strata before cleaning	Number of samples scrub cleaning	Number of samples microfibre cleaning	Mean log_10_ reduction after scrub cleaning (95% CI)	Mean log_10_ reduction after microfibre cleaning (95% CI)	*p*-value
Floor	< 5.0 CFU/cm^2^	10	6	0.48a (0.02–0.93)	-0.08a (-0.57-0.40)	0.063
Floor	5.0–40.0 CFU/cm^2^	9	14	1.07b (0.76–1.37)	0.32a (-0.04-0.68)	0.0022
Floor	> 40.0 CFU/cm^2^	4	3	1.46b (0.01–2.91)	1.70b (-1.19-4.59)	0.78
Wall	< 5.0 CFU/cm^2^	16	15	0.86a (0.56–1.17)	0.40a (0.17–0.63)	0.016
Wall	≥ 5.0 CFU/cm^2^	7	8	2.13b (1.45–2.82)	0.57a (0.30–0.83)	0.00085

Compact letter display (a and b) show if mean log_10_ reduction within cleaning method and surface differ or not between strata

### Effect of cleaning, comparisons within cleaning method

Scrub cleaning of floors and walls with a bacterial load above or equal to 5.0 CFU/cm^2^ before cleaning resulted in higher bacterial reduction compared to surfaces with a bacterial load below 5.0 CFU/cm^2^, see Table 3. Microfibre cleaning of the floors with a bacterial load above 40 CFU/cm^2^ resulted in higher bacterial reduction compared to cleaning of floors with a bacterial load below or equal to 40.0 CFU/cm^2^.

### Effect of disinfection

On the floors, the mean bacterial reduction after disinfection was 0.68 log_10_ in cages that were microfibre cleaned, compared to 0.21 log_10_ in cages that were scrub cleaned (p-value 0.015). The equivalents on the walls were 1.21 log_10_ respectively 0.037 log_10_ (p-value 2.3e-5). In four cases there were a ~ 1 log_10_ increase in bacterial load after disinfection on scrub cleaned walls, from ~ 0.05 CFU/cm^2^ to ~ 0.6 CFU/cm^2^.

### Relationship between bacterial load on the floor and observed explanatory factors

**Fig. 5:**
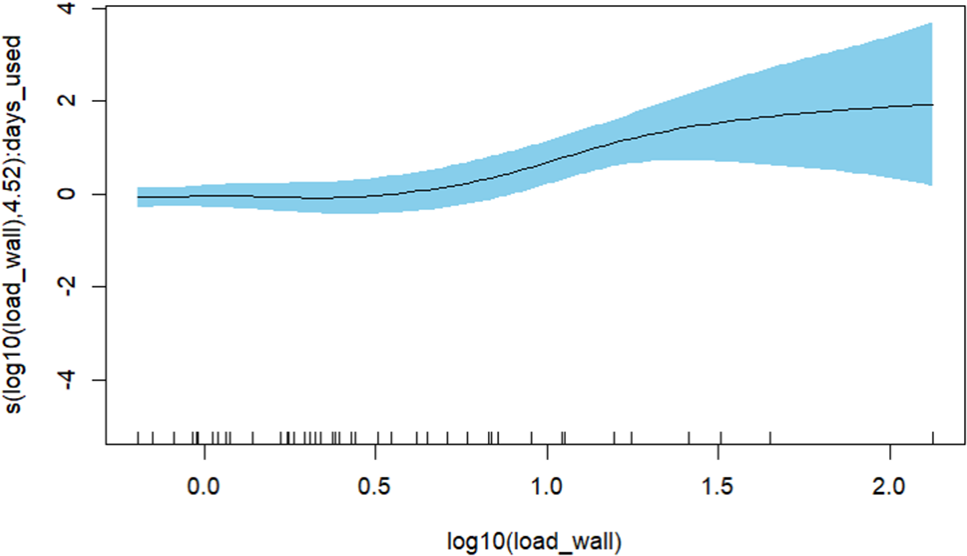
The relationship between the bacterial load on the floors before cleaning and the bacterial load on the walls before cleaning and time patient spent in the cage. The y-axis (days) shows the spline effect (s) of the interaction of the log_10_-transformed bacterial load on the wall by time patient spent in the cage. The x-axis shows the bacterial load on the walls in log_10_ CFU/cm^2^. X-values are plotted along the bottom of the plot. The full line shows the nonlinear relationship (edf:4.52) between the time the patient spent in the cage (the interaction of the log_10_-transformed bacterial load on the wall by time patient spent in the cage) and the log_10_-transformed bacterial load on the wall. The blue shade shows the 95% confidence interval for the mean shape of the effect

**Fig. 6:**
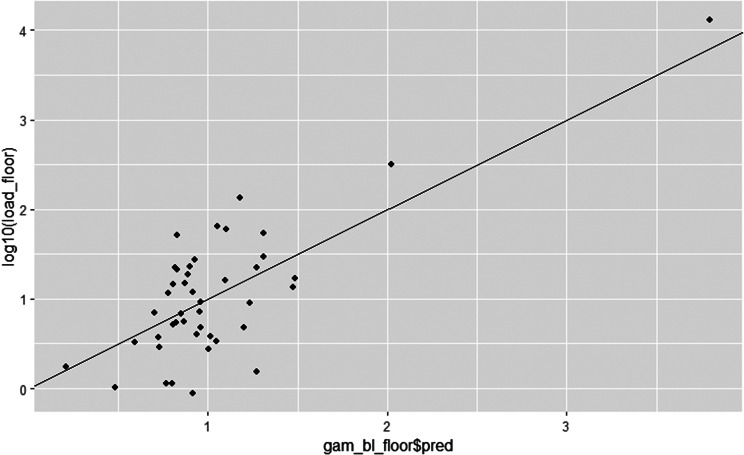
Relationship between the bacterial load on the floors and predicted bacterial load on the floors. The y-axis (days) shows the bacterial load on the floor, in log_10_ CFU/cm^2^, from the original data set. The x-axis shows the predicted bacterial load on the floors in log_10_ CFU/cm^2^. The full line is a regression line going through the intercept (0) with a slope of 1

Spearman’s rank correlation showed no correlation between the bacterial load on the floors and the bacterial load on the walls before cleaning. The relationship between the bacterial load on the floors before cleaning and time patient spent in the cage (average = 1.66 days), time the cage was empty before cleaning (average = 1.66 days) and the bacterial load on the walls was investigated using a GAM model. The GAM model as a whole explained 58.7% of the bacterial load on the floor before cleaning. The interaction of the bacterial load on the walls by time patient spent in the cage contributed significantly to the bacterial load on the floors (*p* = 0.004). The bacterial load increased with longer stay in the cage. The interaction of the bacterial load on the wall by time the cage was empty before cleaning contributed to some extent to the bacterial load on the floors, since the deviance explained by the GAM model increased when the interaction was included, but not significantly (*p* = 0.12). There was a tendency that the bacterial load declined with longer empty time before cleaning. The relationships between the bacterial load on the floors and the interaction of the bacterial load on the walls by time patient spent in the cage respectively the interaction of the bacterial load on the wall by time the cage was empty before cleaning were nonlinear, see Fig. 5 and Additional file 1. The prediction of bacterial load on the floors was rather accurate when the bacterial load was between 0.5 and 1.5 log_10_ CFU/cm^2^, see Fig. 6. When the bacterial load was below 0.5 log_10_ CFU/cm^2^ or above 1.5 CFU/cm^2^ the model both under- and overestimated the bacterial load on the floors.

## Discussion

The bacterial load before cleaning was higher on both the floors and the walls after a longer stay in the cage. This is no surprise as the contact-time with the surface was longer. This can be compared with the results by Adams et al. [27] where surface contamination was higher on surfaces often touched and most of the isolated *Staphylococcus aureus* were from surfaces with a high total bacterial load. Based on this it can be assumed that a longer stay in a cage also may increase the amount of bacteria into the environment and then indirectly spread to patients. The bacterial load was higher on the floors compared with the walls, which can probably be explained by more direct patient contact with the floor, which is often soiled with body fluids and food particles, or bedding material on the floor and that the anti-slip surface on the floor is difficult to clean and disinfect. The bacterial load on cage walls was rather low before cleaning and most of the samples after cleaning, with both methods, were below 2.5 CFU/cm^2^ indicating the walls are less contaminated and therefore easier to effectively clean than the floors. The results from this study show that cage floors, with an anti-slip surface, in animal healthcare facilities must be considered as a risk surface for transmission of bacteria between patients if cleaned and disinfected improperly.

Scrub cleaning was generally more effective in reducing the bacterial load on the surfaces cleaned in our study, compared to microfibre cleaning. Plausible explanations for this are the scrub cleaning’s cleaning qualities on the surface. Rinsing before cleaning may rinse away loose particles and will wet the surface which may be beneficial in the cleaning process. Even though the surface material on floors in human healthcare are generally easier to clean and manual scrub cleaning is not the same as using a scrubbing machine the result is, to some extent, comparable with a study where wet scrubbing, with a scrubbing machine, was the most effective cleaning method to reduce coagulase-positive staphylococci on floors in human healthcare [20]. Damp microfibre cloths has been reported to have good cleaning effect on laminate, steel, smooth (vinyl) and rough (linoleum) tiles [16] but anti-slip surfaces are rougher and it is reasonable that scrub cleaning, due to the better mechanical cleaning effect from scrubbing, is more effective compared to damp microfibre mops on such surfaces. The use of detergent in this study may have increased the cleaning effect as it did in the study by Robertson et al. [17]. Rinsing may reduce the bacterial load even further on cleaned surfaces since bacteria that has come off from the surface but not stuck on the cleaning equipment is rinsed from the surface.

Disinfection resulted in a higher bacterial reduction in cages cleaned with microfibre compared to those that were scrub cleaned. This can be explained by the fact that there were more bacteria left on the surfaces after microfibre cleaning, i.e. more bacteria to eliminate.

In this study, the majority of the samples yielded below 2.5 CFU/cm^2^ after cleaning, except for samples from the microfibre cleaned floors where 65% showed a bacterial load ≥ 2.5 CFU/cm^2^ and 39% ≥ 5.0 CFU/cm^2^. A bacterial load below 2.5 CFU/cm^2^ after cleaning can be considered a good result as studies from animal and human healthcare have shown that pathogenic bacteria are often found on surfaces with a high total bacterial load [4, 27]. With more knowledge of the effect of environmental cleaning and disinfection in animal healthcare facilities, it would be relevant to perform a risk assessment locally to inter alia decide if there are risk sites that should be disinfected after cleaning. A relevant threshold value for total bacterial load to prevent environmental spread of bacteria in animal healthcare is not known, but low bacterial loads can be assumed to reduce the risk of environmental spread of most bacteria, including pathogenic bacteria and secondary HAIs.

An increase in bacterial load on the floors after microfibre cleaning was noted at one occasion. This could be caused by a disrupted biofilm, as was proposed to be the cause of increased detection of multidrug-resistant organisms in floor drains after cleaning, in a study in human healthcare [28]. In human healthcare dry surface biofilms have been found on 83–95% of detergent cleaned and chlorine disinfected surfaces [29–31]. When sampling a surface with a biofilm there is a risk the bacterial load on the surface will be underestimated, since the bacteria is protected in the biofilm and bacteria in biofilms are difficult to culture [32, 33]. Cleaning and disinfection are often incapable to eliminate biofilm, which may pose a risk to the next patient since bacteria may be left on the surface and bacteria in biofilms may carry, share and spread resistant genes [34]. The occurrence of dry surface biofilm was not evaluated in the present study but it could be relevant for future studies of the effect of environmental cleaning and disinfection in animal healthcare.

A limitation in the study was that only cages that were visibly clean or with sporadic soiling were included in the study. But, it enabled standardization of the cleaning protocol for each surface. The cleaning time per surface was set to 60 ± 10 s, meaning 4 min ± 40 s was spent on cleaning each cage, a cleaning time that likely can be considered reasonable even if shorter cleaning time is common in animal healthcare settings. Another limitation was that the used program in the washer-disinfector did not include cleaning before disinfection. Since the scrubbing brush was only used for cleaning one cage, then rinsed with tap-water outside the cage directly after use and finally rinsed in the washer-disinfector it is likely that the scrubbing brush was clean enough for the heat disinfection to effectively eliminate microorganisms. The reduced bacterial load after scrub cleaning also indicates that the scrubbing brushes were clean enough so contamination from them to the surface could be neglected.

## Conclusion

Overall the bacterial load was below suggested threshold values after decontamination, except after microfibre-cleaning of the floor. Scrub cleaning with a detergent should be considered for cleaning of anti-slip surfaces like the cage floor. There is a need for evidence-based cleaning and disinfection routines for near-patient surfaces and evidence-based threshold values for bacterial load, to reduce the risk of HAIs.

## Electronic supplementary material

Below is the link to the electronic supplementary material.

Supplementary Material 1

## Data Availability

The datasets used and analysed during the current study are available from the corresponding author upon a reasonable request.
