# Supplementary material for: A comparison of two cleaning methods applied in a small animal hospital
Source: BMC Vet Res. 2025 Mar 15;21:171. doi: 10.1186/s12917-025-04631-0 (PMC11910008; doi:10.1186/s12917-025-04631-0)
Supplement: Supplementary file 1 — Supplementary Material 1 [file 12917_2025_4631_MOESM1_ESM.pdf]

Additional file 1: relationship between the bacterial load on the floors before cleaning and the bacterial load on the walls before cleaning and time the cage was empty before cleaning

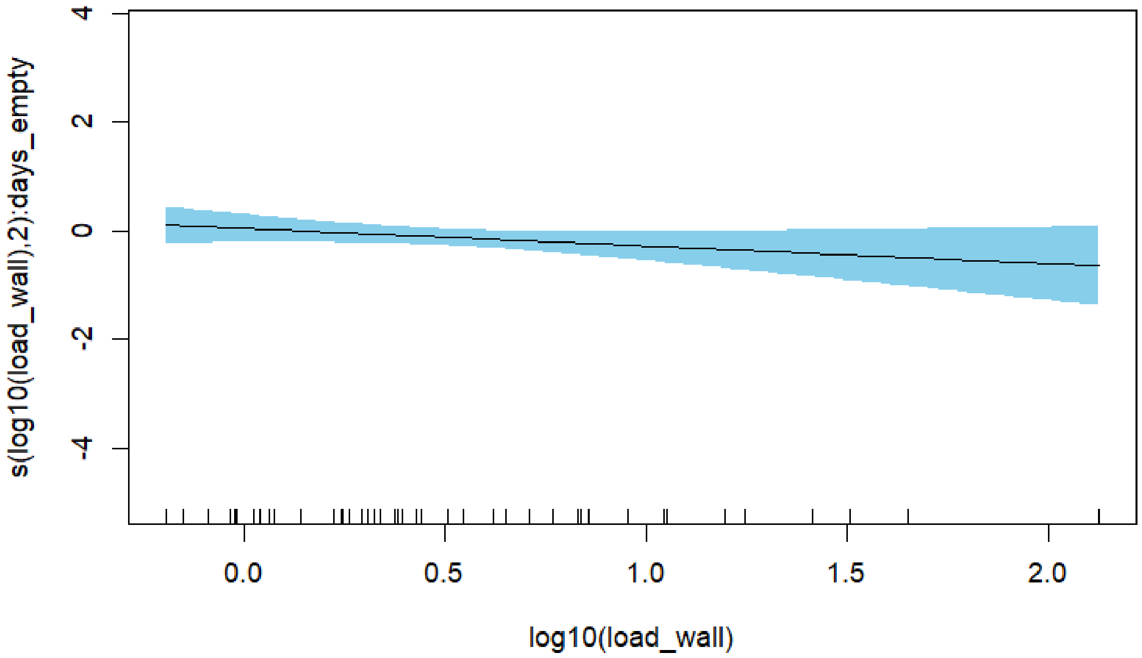

The relationship between the bacterial load on the floors before cleaning and the bacterial load on the walls before cleaning and time the cage was empty before cleaning. The y-axis (days) shows the spline effect (s) of the interaction of the log_{10}-transformed bacterial load on the walls by the time the cage was empty before cleaning. The x-axis shows the bacterial load on the walls in log_{10} CFU/cm^2. X-values are plotted along the bottom of the plot. The full line shows the nonlinear relationship (edf:2) between the time the cage was empty before cleaning (the interaction of the log_{10}-transformed bacterial load on the wall by time the cage was empty before cleaning) and the log_{10}-transformed bacterial load on the wall. The blue shade shows the 95% confidence interval for the mean shape of the effect.
